# Murine gammaherpesvirus-68 expands, but does not activate, CD11b^+^ gr-1^+^ splenocytes in vivo

**DOI:** 10.1186/1476-9255-9-14

**Published:** 2012-04-16

**Authors:** Daniel A Nelson, Vinita S Chauhan, Melanie D Tolbert, Kenneth L Bost

**Affiliations:** 1 Department of Biology, University of North Carolina at Charlotte, 9201 University City Blvd., Charlotte, NC, 28223, USA

**Keywords:** Myeloid derived suppressor cells, Gammaherpesvirus

## Abstract

**Background:**

Murine gammaherpesvirus 68 (HV-68) is an efficient pathogen, capable of infecting and establishing lifelong latency in rodents. While many studies have demonstrated the ability of this viral infection to modulate immune responses, a unifying mechanism for HV-68-induced subversion of a protective host response remains elusive. We questioned whether infection with HV-68 could expand a population of myeloid derived suppressor cells (MDSC) as one mechanism for altering protective immunity.

**Methods:**

Mice were infected with HV-68, with viral latency being established in these animals. At varying times post-infection, cells were isolated for detection of viral genomes, phenotyping of myeloid cell populations, and ex vivo analysis of suppressor activity of myeloid cells.

**Results:**

CD11b + Gr-1+ myeloid cells accumulated in the spleens, but not the bone marrow, of HV-68 infected mice. These cells were predominantly Gr-1+ Ly-6 G+, and could be found to contain viral genomes. Increased levels of serum S100A8/A9 produced during viral infection were consistent with the expansion of these CD11b + Gr-1+ myeloid cells. Despite their expansion, these cells exhibited no increased arginase 1 or iNOS activity, and did not have the ability to suppress anti-CD3 antibody activated T lymphocyte responses.

**Conclusions:**

We concluded that HV-68 infection was capable of expanding a population of myeloid cells which were phenotypically similar to MDSC. However these cells were not sufficiently activated during the establishment of viral latency to actively suppress T cell responses.

## Introduction

Myeloid-derived suppressor cells (MDSC) are hematopoietic precursors of macrophages, granulocytes, and dendritic cells [1]. These immature cells are CD11b + Gr-1+ and can be found in normal bone marrow and lymphoid organs. In response to inflammation [2], sepsis [3], trauma[3], some autoimmune diseases [4], and some cancers [5], the population of MDSC can be dramatically expanded. These cells have been implicated in limiting the immune response, with a particular focus on their ability to suppress T lymphocyte activation [1]. Mechanisms of MDSC suppressive activity include the production of arginase, iNOS, and reactive oxygen or nitrogen species by these cells, as well as their ability to induce T regulatory cells.

Induction of MDSC during microbial infections has also been reported. Using a cecal ligation and puncture model of bacterial sepsis, the presence of MDSC was found to be beneficial for survival [3]. In parasitic infections [6], the expansion of MDSC may represent an attempt by the host to balance harmful inflammation while trying to eliminate the pathogen. In acute influenza A infection, the presence of MDSC was thought to be immunosuppressive [7]. A mouse model of hepatitis B virus infection also suggested that the expansion of MDSC and their immunosuppressive effect might contribute to the chronic nature of this viral disease [8].

In the present study, we report the expansion of CD11b + Gr-1+ cells following infection with murine gammaherpesvirus 68 (HV-68). HV-68 has been used as a model for studying the human gammaherpesviruses, Epstein Barr virusand Kaposi’s sarcoma–associated herpesvirus[9]. This model seems quite useful based on the genetic similarity of HV-68 to the human gammaherpesviruses, and on the similarities in pathologies induced in infected mice. After intranasal or oral inoculation of HV-68 in mice, there is a productive infection of epithelial cells, followed by infection of B lymphocytes, macrophages and dendritic cells in peripheral organs like the spleen. By days 9–11 post infection most of the infectious virus is cleared, and a marked mononucleosis and splenomegaly occurs which peaks around day 15 post-infection. During this time the virus establishes latency, with the long-term presence of the virus maintained throughout the life of the animal. Many studies have indicated that the immune response is altered following the establishment of HV-68 latency in rodent models. For example, chronic inflammatory conditions such as EAE [10], inflammatory bowel disease [11], pulmonary fibrosis [12], atherosclerotic lesions [13,14] are exacerbated in HV-68 infected mice. Such alterations in the inflammatory response could result from one or multiple HV-68mediated alterations in the innate and adaptive immune responses that have recently been reviewed [9]. At present the precise mechanisms, which allow HV-68 to subvert a protective host response and exacerbate inflammatory conditions, are not clear. In the present study, we discovered that HV-68 could induce the accumulation of cells with an MDSC phenotype in the spleens of infected mice.

## Materials and methods

### Animals

Six to eight week old female BALB/c mice (18–22 g) were purchased from Jackson Laboratories (Bar Harbor, ME) and housed in the vivarium in filter top cages containing sterile bedding. After arrival, mice were quarantined for at least five days, and fed chow and water ad libitum. All animal experiments were in compliance with protocols approved by the University of North Carolina at Charlotte Animal Care and Use Committee.

### Maintenance of murine gammaherpesvirus-68 (HV-68) stocks

Murine gammaherpesvirus-68 (HV-68; ATCC # VR-1465) stocks were prepared by infecting baby hamster kidney cells (BHK-21; ATCC # CCL-10) at a low multiplicity of infection, followed by preparation of cellular lysates, as described previously[15-17].Replicating HV-68 was quantified by adding 1:3 serial dilutions of cell media or lysates to BALB/3 T12-3 cell (ATCC # CCL-164) monolayers. After the monolayers were incubated with virus, cells were overlayed with 1% plaqueassay agarose (BD Biosciences, San Diego, CA) in medium with 30% fetal bovine serum. After 5 days in 5% CO_2_, overlays were removed and cell monolayers fixed and stained with crystal violet. All serial dilutions were performed in triplicate.

### Nucleic acid isolation

Five to twenty-five milligrams of tissue or cells were suspended in 250 μl of ice-cold phosphate-buffered saline (PBS) and homogenized briefly in 1.5 ml microfuge tubes. An equal volume of PBS containing 2% SDS, 10 mM EDTA and 50 μg/ml Proteinase K (Sigma-Aldrich, St. Louis, MO) was added and homogenates incubated overnight at 37^o^C. Nucleic acid was extracted 2X with saturated phenol/chloroform/isoamyl alcohol (25:24:1), precipitated with 2 volumes of EtOH, and resuspended in PBS with 25 μg/ml RNase A (Sigma-Aldrich). After incubation for 30 min at 37^o^C, DNA was extracted 1X with phenol/chloroform/isoamyl alcohol, precipitated with 2 volumes ethanol, microfuged for 10 min at 16,000 × g and washed with 75% EtOH. The DNA pellet was air dried and resuspended in 10 mM Tris, pH 8.0. DNA concentration was determined by absorbance at 260 nm.

Total RNA was isolated using Trizol (Invitrogen; Carlsbad, CA), as previously described[15,16,18]. RNA samples were incubated with RNase-free pancreatic DNase (RQ1 DNase, Promega, Madison, WI) as per the manufacturer’s instructions, the RNA precipitated with EtOH and resuspended in 50 μl of nuclease-free H_2_O. RNA concentrations were determined with a Gene Spec III spectrophotometer (Naka Instruments, Japan) using a 10 μl cuvette. For cDNA synthesis, one μg of RNA was reverse-transcribed in the presence of random hexamers (50 ng/μl), 10 mM dNTPs, 2.5 mM MgCl_2_ using ImProm-II reverse transcriptase (Promega) in the buffer supplied by the manufacturer. cDNA was precipitated with one-tenth volume of 3 M sodium acetate (pH 5.2) and 3 volumes of EtOH, and resuspended in 50 μl of nuclease-free H_2_O.

### Semiquantitative and quantitative PCR and RT-PCR

Genomic DNA and mRNA transcript levels were examined by PCR. For semiquantitative PCR, 100 ng of DNA or cDNA was combined with 2.5 U of Taq polymerase (Promega), 0.2 mM each dNTP, 25 pmol of each primer and PCR buffer containing 2.5 mM MgCl_2_ as provided by the manufacturer. Samples were cycled using 95° denaturation for 35 seconds, 60°C annealing for 75 seconds and 72°C extension for 90 seconds, with the first three cycles using extended denaturation, annealing and extension times. PCR was for 35 cycles, except for GAPDH, which was for 28 cycles. The extension time of the last cycle was for 5 min at 72°C. Forty percent of each amplified PCR product was electrophoresed on an ethidium bromide-stained 2% agarose gel and photographed under UV illumination.

For quantitative PCR, mRNA expression was quantified relative to GAPDH expression using an Applied Biosystems 7500 Fast Real-time PCR System (FosterCity, CA) and SYBR Green I for double-stranded DNA detection. Amplifications were performed in a total volume of 10 μl containing QuantiTect SYBR Green PCR Master Mix (Qiagen), primer pairs (0.5 μM), template cDNA (20 ng) and nuclease-free water. Samples were cycled 45 times, starting with an initial activation step of 15 min at 95°C, followed by denaturation for 15 sec at 95°C, annealing for 30 sec at 60°C and extension for 30 sec at 72°C. Data were acquired at 77°C for ORF65 and 80°C for GAPDH for 2 sec after extension.Changes in mRNA expression are presented as mRNA fold-induction, where

Fold-induction = 2_T_^(C^_T_^(Cytokine) Control – C^^(Cytokine) Sample)^/2_T_^(C^_T_^(GAPDH) Control – C^^(GAPDH) Sample)^ and C_T_ = the threshold cycle.

PCR primer sets were designed by using IDT SciTools and purchased from IDT (Integrated DNA Technologies, Coralville, IA). Primer sets used for amplification are as follows:

ORF65(open reading frame-65 - murid herpesvirus 4; accession no. NC_001826; 221 bp product)

Forward: 5’ - ATG CTC CAG AAG AGG AAG GGA CAC - 3’

Reverse: 5’ - TTG GCA AAG ACC CAG AAG AAG CC - 3’

TAAR1(trace amine-associated receptor 1 - accession no.NM_053205; 159 bp product)

Forward: 5’ - ACT TCA ACC CAC CAA CTG GCT - 3’

Reverse: 5’ - AGC ATG ATA TCG GTG CTG GTG TGA - 3’

ORF50 (open reading frame-50 - murid herpesvirus 4; accession no. NC 001826; 355 bp)

Forward: 5’ – ATG GCA CAT TTG CTG CAG AAC - 3’

Reverse: 5’ – ACG GCG CCT GTG TAC TCA A – 3’

HV-68M3 (murid herpesvirus 4; accession no. NC 001826; 275 bp)

Forward: 5’ - TCT CTT GAC CCA GCT CTT CCA CAG - 3’

Reverse: 5’ - AGG AGG ATT GGC TGA GAG CCT TAC - 3’

GAPDH(glyceraldehyde-3-phosphate dehydrogenase; accession no. NM_008084; 346 bp - spans exons 3to 5)

Forward: 5’ - CCA TCA CCA TCT TCC AGG AGC GAG - 3’

Reverse: 5’ –CAC AGT CTT CTG GGT GGC AGT GAT-3’

### HV-68 infection of mice, isolation of cells from tissues,and FACS analyses

Groups of mice were anesthetized with isoflurane and mock treated by intranasal instillation of saline, or infected intranasally with 6 × 10^4^plaque forming units of HV-68 as previously described [15-17]. At the indicated days post-infection, mice were euthanized and tissues excised. Bone marrow cells were isolated from the long bones of individual mice as previously described [16,18,19]. Splenocyte single cell suspensions from individual mice were made by passing tissue through a 30-gauge wire mesh. Total spleen cells and bone marrow cells were washed with sterile PBS (300 × g for 10 min), resuspended in PBS containing 10% non-immune rabbit serum (Invitrogen, Camarillo, CA) and incubated on ice for 45 min with antibodies conjugated with appropriate fluorochromes for multiparameter staining (anti-CD11b, anti-Gr-1, anti-Ly-6 G, and anti-Ly-6 C; eBioscience, San Diego, CA).After washing, cells were resuspended, analyzed, and then sorted using the FACSAria II cell sorter (BD Biosciences,San Diego, CA). Analyses were performed on >20,000 cells per individual spleen or bone marrow isolate. Cell subpopulations (CD11b+, CD11B-, CD11B + Gr-1+, CD11B- Gr-1-, Gr-1+ Ly-6 G+, and Gr-1+ Ly-6 C+) from individual mice were sorted and cells pelleted at 1000 × g for nucleic acid isolation.

### S100A8 and S100A9 ELISA

Mice were uninfected or infected with HV-68 as described above. At the indicated times post infection, mice were euthanized and individual sera were taken. The total amount of S100A8 and S100A9 in each serum sample was determined using an ELISA capable of detecting both proteins (ALPCO, Inc., Salem, NH).

### Arginase 1 and iNOS activity

CD11b^+^ Gr-1^+^ cells were isolated using a mouse Myeloid-Derived Suppressor Cell Isolation Kit (Miltenyi Biotec, Auburn, CA). The isolated cells were cultured for 24 hr in tissue culture medium (RPMI plus 10% fetal bovine serum) and supernatants used for iNOS assays and cells for arginase 1 assays.

The activity of arginase 1 was determined as follows: Cells were lysed in 100 μl of 0.1% Triton X-100 for 30 min at 37°C, and microfuged to remove debris. 100 μl of 25 mM Tris–HCl (pH 7.4) and 10 μl of 10 μM MnCl_2_ were added to the supernatant and incubated for 10 min at 56 °C. 100 μl of 0.5 M L-arginine (pH 9.5) was added and incubate at 37°C for 30 min. The reaction was stopped by the addition of 900 μl of 96% H_2_SO_4_, 85% H_3_PO_4_ and H_2_O (1/3/7) and then 40 μl of α-isonitrosopropiophenone (4% in EtOH) was added. Samples were heated at 95°C for 30 min along with serially diluted urea standards. The absorbance was read at 570 nm.

The activity of iNOS was determined using the Griess Reagent System (Promega, Madison, WI).

### MDSC isolation from HV-68 infected mice and co-culture with activated T lymphocytes

For MDSC isolation, groups of mice were mock treated or infected with HV-68. At 15 days post infection, mice were euthanized and spleens were removed. Single cell suspensions of splenocytes were made as described above, and red blood cells were removed using a lysing reagent (Sigma-Aldrich, St. Louis, MO). Splenic leukocytes were washed and resuspended in RPMI-1640 containing 10% fetal bovine serum. CD11b + Gr-1 + cells were then isolated using a mouse MDSC isolation kit (Miltenyi Biotec, Auburn, CA).

To isolate T lymphocytes, spleens were removed from normal, uninfected mice, and splenic leukocytes obtained as described above. Total T lymphocytes were isolated using magnetic separation (Pan T cell Isolation Kit; Miltenyi Biotec).

For co-cultures, the indicated numbers of MDSC were added to 2 X 10^5^ T lymphocytes in anti-CD3 coated microtiter wells (T cell activation plates, BD Biosciences, Bedford, MA). After 72 hours of co-incubation, the amount of IFN-γ present in the culture supernates was determined using an ELISA (DuoSet mouse IFN-γ; R&D Systems, Minneapolis, MN) as an indication of T lymphocyte activation.

### Statistics

For statistical analysis, data were analyzed using GraphPad Prism 5 software (GraphPad Software, Inc., San Diego, CA). Analyses were performed using Student’s *t*-test, or by one-way analysis of variance (ANOVA) with Tukey’s Multiple Comparison Test as post-test. Mean values are presented in the figures +/− the standard error of the mean (SEM). Results marked with an asterisk (*) were determined to be statistically significant at P < 0.05.

## Results

### Increased percentage of splenic CD11b^+^ gr-1^+^ cells during the leukocytosis phase of HV-68 infection

**Figure 1:**
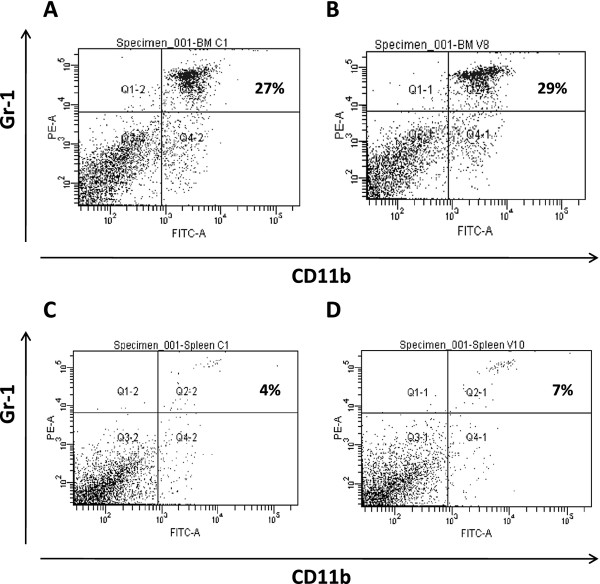
**Increased percentage of splenic CD11b**^**+**^**Gr-1**^**+**^**cells during the leukocytosis phase of HV-68 infection.** Groups ofBalb/c mice were mock-treated or intranasally infected with HV-68 virus. During the peak of viral-induced leukocytosis (15 days post-infection), bone marrow cells and splenocytes were isolated from individual animals and labeled with fluorchrome-conjugated anti-CD11b and anti-Gr-1 antibodies. FACS analysis of one representative animal per group shows the percentage of CD11b^+^ Gr-1^+^ cells (upper right quadrant) from thebone marrow (Panel **A**) or spleen (Panel **C**) of a mock-treated mouse, or from the bone marrow (Panel **B**) or spleen (Panel **D**) ofan HV-68 infected mouse.

HV-68 infection results in a mononucleosis-like expansion of leukocytes which peaks around 15 days post infection. We questioned whether the number and percentage of CD11b + Gr-1+ cells increased during this leukocytosis. Figure 1 shows representative FACS analyses of bone marrow cells and splenocytes of one mock and one infected animal. No significant difference was observed in the percentage of CD11b + Gr-1+ in the bone marrow of these mice (Figure 1A, 27% versus 29%). However, there was a significant increase in the percentage of CD11b + Gr-1 + cells in the spleens of mice 15 days following infection (Figure 1B, 4% versus 7%). In HV-68 infected mice, there is a splenomegaly resulting from the mononucleosis-like leukocytosis. This leukocytosis results in an increase in the total splenic leukocytes isolated from mock (e.g. 6.2 X 10^7^ ± 1.1 X 10^7^) versus infected (e.g. 1.8 X 10^8^ ± 1.8 X 10^7^) animals. Therefore, not only is the percentage of CD11b + Gr-1+ cells in the spleens of HV-68 infected mice higher, but the absolute number of these cells is also increased during the mononucleosis-like disease.

**Figure 2:**
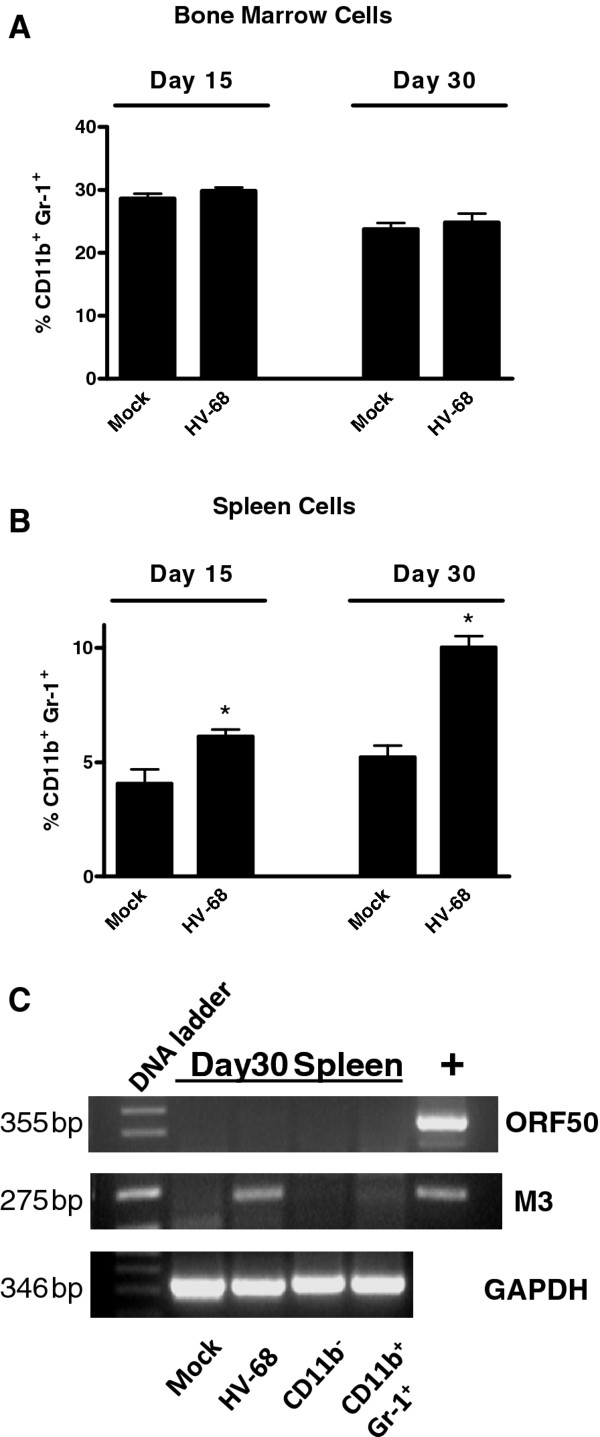
**Increased percentage of splenic CD11b**^**+**^**Gr-1**^**+**^**cells during leukocytosis phase of HV-68 infection and during viral latency.** Groups of Balb/c mice were mock-treated or intranasally infected with HV-68 virus. During the peak of viral-induced leukocytosis (15 days post-infection) or after viral latency had been established (30 days post-infection), bone marrow cells and splenocytes were isolated from individual animals and labeled with fluorchrome-conjugated anti-CD11b and anti-Gr-1 antibodies. FACS analysis was performed to determine the percentage of CD11b^+^ Gr-1^+^ cells in each cell population. Results are presented as mean values (N = 5) ± standard errors for bone marrow cells (Panel **A**) or spleen cells (Panel **B**).Asterisks indicate a statistically significant difference (p < 0.05) when comparing Mock versus HV-68 infected mice. These studies were performed three times with similar results. To demonstrate viral latency in mice infected for 30 days (Panel **C**), RNA isolated from spleens was subjected to RT-PCR to detect expression of the M3 RNA (a latent transcript) and the lack of expression of ORF 50 RNA (a replicating viral transcript). Representative results of one animal (N = 5) are presented as amplified products electrophoresed on ethidium bromide-stained gels. Positive controls (+) for ORF50 and M3 amplifications are shown in the last lane. Presence of GAPDH mRNA expression in each RNA sample using RT-PCR was also performed as a positive control.

Figure 2 shows cumulative data from 5 different mock and HV-68 infected mice. No significant differences in the percentage of CD11b + Gr-1+ in the bone marrow were observed (Figure 2A). However, significant increases in the percentage of CD11b + Gr-1+ in the spleens of infected mice were observed (Figure 2B). Importantly, this difference was seen at day 15 post infection, but also at day 30 post-infection when leukocytosis was diminished. While the latent viral transcript, M3, could be detected using RT-PCR analyses in spleens, the lytic viral transcript, ORF50, could not be detected (Figure 2C). Therefore increased percentages and numbers of CD11b + Gr-1+ cells that accumulate during the mononucleosis phase of the disease, persist in the spleens of latently infected mice.

### Fractionation of CD11b + Gr1+ spleen cells into ly-6 G + and ly-6 G- populations

**Figure 3:**
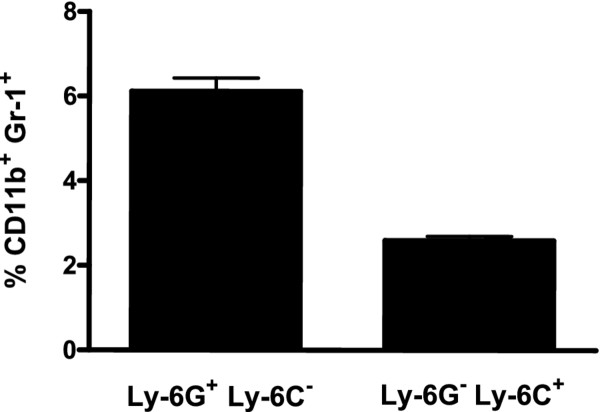
**Fractionation of CD11b + Gr1+ spleen cells into Ly-6 G + and Ly-6 G- populations.** Groups of Balb/c mice were intranasally infected with HV-68 virus, and at 15 days post-infection splenocytes were isolated from individual animals. CD11b + Gr1+ cells were isolated from these splenocytes, and labeled with fluorchrome-conjugated anti-Ly-6 G and anti-Ly-6 C antibodies. FACS analysis was performed to determine the percentage of each cell population. Results are presented as mean values (N = 5) ± standard errors. These studies were performed three times with similar results.

To further define the phenotype of HV-68-expanded CD11b + Gr1+ cells in the spleens of infected mice, these cells were isolated and stained for the presence of Ly-6 G and Ly-6 C. As shown in Figure 3, the majority of these cells were Gr-1+ Ly-6 G+, with expansion of Gr1+ Ly-6 C + cells being less frequent.

### CD11b + gr-1+ cells from spleens of HV-68 infected animals harbor HV-68 viral genomes

**Figure 4:**
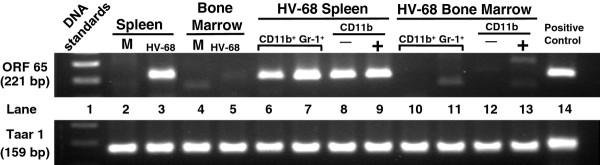
**CD11b + Gr-1+ cells from spleens of HV-68 infected animals harbor HV-68 viral genomes.** Groups of Balb/c mice were mock-treated (M) or intranasally infected with HV-68 virus (HV-68). During the peak of viral-induced leukocytosis (15 days post-infection), bone marrow cells and splenocytes were isolated from individual animals and unfractionated (Spleen, Bone Marrow) or fractionated into CD11b positive (CD11b+), CD11b negative (CD11b-), or CD11b positive plus Gr1 positive (CDb + Gr-1+) cell populations. DNA was isolated from each cell fraction and assayed for the presence of viral ORF65 by PCR. Representative results of one set of cell fractionations (N = 3) are presented as amplified products electrophoresed on ethidium bromide-stained gels. The presence of the Taar1 gene in each DNA sample using PCR was also performed as a control for DNA loading in each sample

To question whether CD11b + and CD11b- populations of cells harbored viral genomes, PCR for the DNA encoding HV-68 ORF 65 was performed. HV-68 ORF 65 DNA was detected in whole spleen (Figure 4, lane 3), but not in bone marrow cells (Figure 4, lanes 5, 10, 11, 12, and 13). Splenic CD11b + Gr1+ (Figure 4 lanes 6 and 7), CD11b- (Figure 4 lane 8), and CD11b + (Figure 4 lane 9) cells also contained detectable levels of HV-68 ORF 65 DNA.

### HV-68 infection induces S100A8 and S100A9 production in vivo

**Figure 5:**
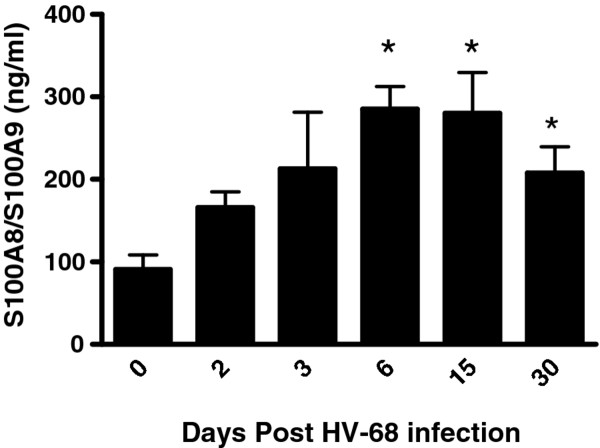
**HV-68 infection induces S100A8 and S100A9 production in vivo.** Groups of mice were untreated (0) or infected with HV-68(HV-68). At the indicated days post-infection sera was taken and assayed for the presence of S100A8 and S100A9 using an ELISA capable of recognizing either protein. Results are presented as means (N = 6) ± standard errors, with asterisks indicating a statistically significant difference (p < 0.05) when comparing untreated versus HV-68 infected mice.

Increased levels of S100A8 and S100A9 could be one factor responsible for the increased number of CD11b + Gr1+ cells in HV-68 infected mice [20]. To address this possibility, serum levels of these proteins were quantified at varying times following infection. Figure 5 shows increasing serum levels of S100A8/S100A9 following infection with HV-68 which is consistent with the observed expansion of CD11b + Gr1+ cells.

### CD11b + gr-1+ cells from spleens of HV-68 infected animals demonstrated no increase in arginase or iNOS activity

**Figure 6:**
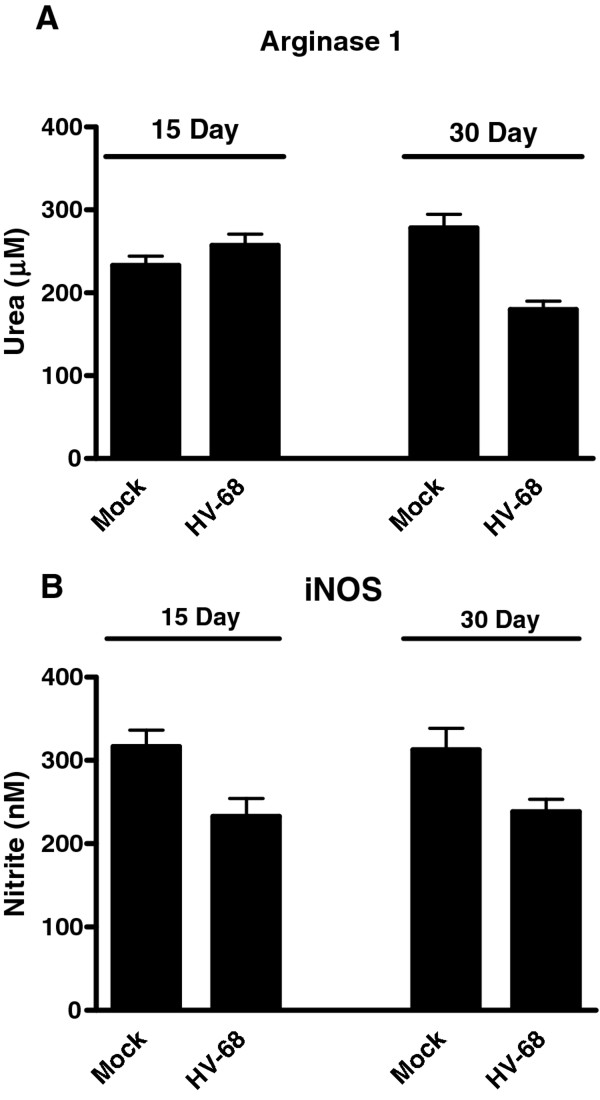
**CD11b + Gr-1+ cells from spleens of HV-68 infected animals demonstrated no increase in arginase or iNOS activity.** Groups of mice were mock treated (M) or infected with HV-68 (HV-68). During the peak of viral-induced leukocytosis (15 days post-infection) or after viral latency had been established (30 days post-infection), CD11b + Gr-1+ splenocytes were isolated from individual animals. Arginase activity and iNOS production by these cells were determined. Results are presented as means (N = 3) ± standard errors.

While it was clear that HV-68 infection increased the number of CD11b + Gr1+ splenocytes, it could not be assumed that these cells had suppressive abilities attributed to MDSC. Increased arginase 1 and iNOS activity can contribute to the suppressive ability of some MDSC[1]. Therefore we compared the levels of enzymatic activity in CD11b + Gr-1+ spleen cells isolated from mock treated or HV-68 infected mice. Figure 6 shows that there was no increase in arginase 1 (Figure 6A) or iNOS (Figure 6B) activity when comparing baseline activity detected in mock treated animals to mice infected with HV-68 for 15 or 30 days, respectively.

### CD11b + gr-1+ cells from spleens of HV-68 infected animals could not suppress anti-CD3 induced T cell activation

**Figure 7:**
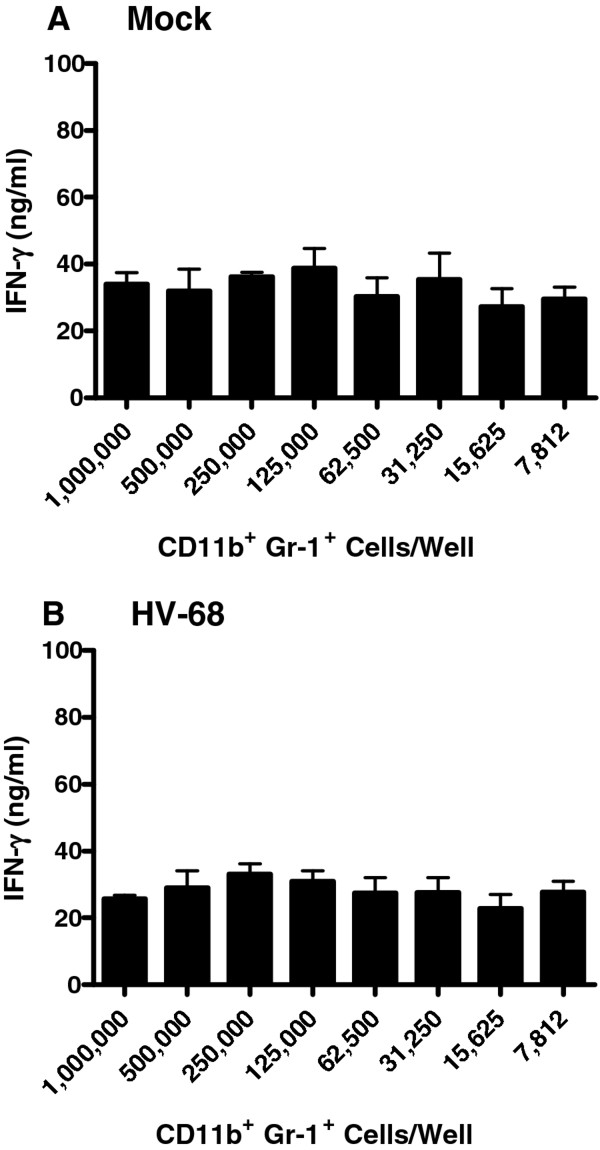
**CD11b + Gr-1+ cells from spleens of HV-68 infected animals could not suppress anti-CD3 induced T cell activation.** Groups of mice were mock treated (Panel **A**) or infected with HV-68 (Panel **B**). During the peak of viral-induced leukocytosis (15 days post-infection)CD11b + Gr-1+ cells were isolated from spleens of individual mice. The indicated numbers of CD11b + Gr-1 + cells were added to 2 X 10^5^cultured T lymphocytes stimulated with anti-CD3 antibodies. After 72 hours of co-incubation, culture supernatants were assayed for interferon gamma (IFN-γ) production as a measure of T cell activation. Results are presented as means (N = 3) ± standard errors.

To further investigate any potential suppressive ability of HV-68-expanded CD11b + Gr-1+ splenocytes, T cell activation studies were performed. CD11b + Gr-1+ splenocytes were isolated from mock treated mice or from mice infected with HV-68 for 15 days. Limiting dilutions of these cells were cultured with2 X 10^5^ T lymphocytes stimulated in anti-CD3 antibody coated microtiter wells. Neither CD11b + Gr-1+ splenocytes isolated from mock treated (Figure 7A) or HV-68 infected mice (Figure 7B) could limit anti-CD3 antibody-induced T lymphocyte IFN-γ secretion.

## Discussion

HV-68 is an efficient pathogen, capable of infecting and establishing lifelong latency in greater than 95% of exposed rodents [21]. Clearly, this virus has evolved mechanisms to limit a protective host response capable of clearing acute or latent virus. Furthermore, increasing the initial inoculum of virus does not result in dramatic increases in pathophysiology, acute-virus mediated death, or latent viral burden [21]. This fact also supports the notion that HV-68 can control or limit the early host response [22], favoring events which ultimately lead to lifelong latency. It has been postulated that the dramatic mononucleosis-like disease which develops, then resolves, during the first 2–3 weeks post infection facilitates the establishment of this viral latency [9].

Mechanisms responsible for limiting protective immunity against HV-68 are not altogether clear. The initiation of a relatively meager pro-inflammatory response during the first few days post-infection has been noted [22], and a listing of HV-68 induced effects on the innate and adaptive immune response has recently been reviewed [9]. Despite these numerous studies a unifying mechanism for HV-68-induced subversion of a protective host response remains elusive.

To further our understanding of how HV-68 infection might limit a protective immune response following infection, we analyzed the expansion of leukocytes during the mononucleosis-like phase of the disease.In a previous publication, we demonstrated that CD11b + cells were increased following HV-68 infection, and that this population of cells could harbor virus [23]. However we did not address whether this population of cells had a phenotype or an activity consistent with an MDSC-induced population.

Here we show that CD11b + Gr-1+ cells are expanded in the spleens, but not bone marrow, of mice infected with HV-68, consistent with the phenotype of MDSC (Figures. 1 and 2). Approximately two thirds of these cells had a Gr1+ Ly6G + phenotype, giving these cells a granulocytic MDSC phenotype [1], whereas the remainder have a monocytic MDSC phenotype, Gr1+ Ly6G- Ly6C + (Figure 3). Of particular interest was the finding that the percentage of MDSC remained expanded for at least 30 days post-infection (Figure 2B). In these mice, no lytic viral transcripts (e.g. ORF50) could be detected, but latent viral transcripts (e.g. M3) were readily present (Figure 2C). We concluded that HV-68 expanded MDSC were present long after acute virus had been cleared from these mice, and that MDSC continued to be present during viral latency. The persistence of increased numbers of MDSC during latency (Figure 2B) could be due to the long-lived nature of MDSC, as has been suggested [24]. Alternatively, mechanisms resulting from infection with HV-68 may contribute to their continued presence.

There are at least two mechanisms by which HV-68 infection could induce MDSC expansion. First, these CD11b + Gr1+ cells could be infected, and the interaction of virus with pattern recognition receptors, or the induction of intracellular signaling pathways (e.g. NF-kB), might contribute to MDSC expansion[1]. Clearly CD11b + Gr1+ cells harbor viral DNA (Figure 4). Future studies will be required to define the percentage of MDSC which are infected and the signaling pathways which might contribute to increasing cell numbers. Second, HV-68 infection could induce the secretion of soluble factors which have been documented to increase MDSC numbers[1]. Based on previous studies, factors such as IL-6 [25], IL-12 [18], and some chemokines [22]are unlikely to make a significant contribution, since these proteins are not prominently expressed in vivo following infection. However other factors such as S100A8 and S100A9 can induce expansion of MDSC, and it was not known whether HV-68 infection could induce systemic expression of these molecules. S100A8 and S100A9 are calcium-binding proteins that form noncovalent homodimers and a heterodimer (S100A8/A9) in a calcium-dependent manner[26]. These proteins have been shown to be critically important for the accumulation of MDSC in cancer models [20,27]. Here we show that following HV-68 infection, serum levels of S100A8 or S100A9 increase during acute infection and are maintained during the establishment of viral latency (Figure 5). The presence of such high levels of S100A8 and S100A9 following infection, and the importance of these proteins for expanding MDSC, suggests one likely mechanism to account for the accumulation of HV-68 induced CD11b + Gr1+ cells in the spleen.

Expansion of MDSC does not necessarily indicate that such cells are activated in a manner that allows them to exert suppressive activity[1]. Mechanisms of MDSC suppressive activity include the production of arginase, iNOS, and reactive oxygen or nitrogen species by these cells, as well as their ability to induce T regulatory cells [1]. Isolated CD11b + Gr1+ from HV-68 infected mice exhibited no increased arginase 1 (Figure 6A) or iNOS activity (Figure 6B) when assayed ex vivo. Furthermore, when dilutions of these cells were co-cultured with anti-CD3 antibody activated T cells, no reduction in IFN-γ production was observed (Figure 7). We concluded from these studies that while HV-68 infection expanded a CD11b + Gr-1+ population of spleen cells, these cells were not fully activated to a suppressor phenotype.

In summary, HV-68 infection can expand a splenic MDSC population, however these cells are not sufficiently activated to function as suppressors. Therefore it is unlikely that these expanded CD11b + Gr-1+ cells make a significant contribution toward limiting a protective anti-viral immune response. Theoretically, however, if HV-68 infected mice were exposed to an additional inflammatory insult which could activate these MDSC, this expanded and activated cell population might contribute to clinical disease. Future studies will address the possibility that HV-68 infected mice might exacerbate disease in cancer models via this expanded CD11b + Gr-1+ cell population where activated MDSC contribute significantly to tumor burden and metastasis.

## Abbreviations

HV-68, Murine gammaherpesvirus-68; MDSC, Myeloid-derived suppressor cells.

## Competing interests

The authors declare that they have no competing interests.

## Authors’ contributions

DAN, VSC, MDT, and KLB each performed selected analyses, each contributed at some level to the study design, and each help to facilitate writing of the manuscript. All authors read and approved the final manuscript.
